# BRDT promotes ovarian cancer cell growth

**DOI:** 10.1038/s41419-020-03225-y

**Published:** 2020-11-30

**Authors:** Ling Chen, Shang Cai, Jing-mei Wang, Ying-ying Huai, Pei-Hua Lu, Qian Chu

**Affiliations:** 1 Department of Pathology, Nanjing Drum Tower Hospital, The Affiliated Hospital of Nanjing University Medical School, Nanjing, China; 2 Department of Radiotherapy and Oncology, The Second Affiliated Hospital of Soochow University, Suzhou, China; 3 Department of Obstetrics and Gynecology, Affiliated Kunshan Hospital of Jiangsu University, 215300 Kunshan, Jiangsu China; 4 Department of Medical Oncology, Wuxi People’s Hospital of Nanjing Medical University, Wuxi, China

**Keywords:** Oncogenes, Ovarian cancer

## Abstract

Bromodomain testis-specific factor (BRDT) is a member of the bromodomain and extra-terminal (BET) family proteins. Its expression and potential functions in ovarian cancer were examined. We show that BRDT is overexpressed in human ovarian cancer tissues and in established (CaOV3)/primary ovarian cancer cells. However, its expression is low in ovarian epithelial tissues and cells. Significantly, shRNA-induced silencing or CRISPR/Cas9-mediated knockout of BRDT inhibited ovarian cancer cell growth, viability, proliferation and migration, and induced significant apoptosis activation. Conversely, exogenous overexpression of BRDT, by a lentiviral construct, augmented CaOV3 cell proliferation and migration. In CaOV3 cells expression of two key BRDT target genes, polo-like kinase 1 (PLK1) and aurora kinase C (AURKC), was downregulated by BRDT shRNA or knockout, but upregulated with BRDT overexpression. In vivo, xenograft tumors-derived from BRDT-knockout CaOV3 cells grew significantly slower than control tumors in severe combined immunodeficient (SCID) mice. Furthermore, intratumoral injection of BRDT shRNA lentivirus potently inhibited the growth of primary ovarian cancer xenografts in SCID mice. Downregulation of PLK1 and AURKC was detected in BRDT-knockout and BRDT-silenced tumor tissues. Collectively, BRDT overexpression promotes ovarian cancer cell progression. Targeting BRDT could be a novel strategy to treat ovarian cancer.

## Introduction

Despite significant progresses have been achieved to improve the overall survival of ovarian cancer in past decades, it is still one primary cause of female mortality^1^. In the United States alone, the American Cancer Society estimates 22,440 new cases of ovarian cancer and 14,080 related deaths each year^2^. Ovarian cancer is typically diagnosed at late stages, due to the lack of effective screening strategy^3–5^. Standard treatments for newly diagnosed ovarian cancers include surgery and platinum-based chemotherapy^3–5^. Yet, because of the significant resistance and recurrence, researchers are focusing on novel oncogenes and cell signaling pathways essential for cancer progression^3–5^.

Bromodomain testis-specific factor (BRDT) is a member of the bromodomain and extra-terminal (BET) family proteins^6^. BRDT, like other BET family proteins (BRD1–4), epigenetically regulates targeted genes expression through interacting with acetylated lysines^7,8^, critical for normal development and disease (cancer) progression^6^. Recent studies have implied that BET family proteins are key oncogenic proteins, being overexpressed in a number of different cancers^7,8^. Inhibitors of BET proteins have displayed promising efficiency against human cancer cells in vitro and in vivo^9,10^.

BRDT is expressed in testes, regulating the meiotic and post-meiotic genes expression to promote spermatogenesis^11^. Cancer cells have the ability to reactivate the normally silent testis-restricted genes, which are important for cancer progression^6^. Recent studies have shown that BRDT is ectopically activated and expressed in human cancers^6^. It has been proposed that BRDT could be a novel biomarker and a possible therapeutic target for human cancer^6^. Several BRDT-regulated genes, including polo-like kinase 1 (PLK1)^12^ and aurora kinase C (AURKC)^13^, are key oncogenic proteins^6,11^. BRDT expression and potential functions in ovarian cancer have not been studied thus far.

## Materials and methods

### Chemicals and reagents

Cell culture reagents were provided by Gibco Co. (Grand Island, NY). Puromycin, polybrene and other reagents were obtained from Sigma-Aldrich (St. Louis, Mo). All primers, sequences and viral constructs were provided by Shanghai Genechem Co (Shanghai, China). The anti-BRDT antibody was purchased from Sigma-Aldrich (SAB2106423). Antibodies for cleaved-caspase-3 (#9664), cleaved-poly (ADP-ribose) polymerase (PARP) (#5625), cleaved-caspase-9 (#20750), Tubulin (#2125) and PLK1 (#4513) were obtained from Cell Signaling Tech China (Shanghai, China). The anti-AURKC antibody was obtained from Abcam (ab46783). The reagents for RNA assays were provided by Thermo-Fisher Invitrogen (Suzhou, China).

### Human tissues

From a total of six (6) primary ovarian cancer patients, the fresh human ovarian cancer tissues (“Ca”) and para-cancer ovarian epithelial tissues (“S”) were acquired. Tissues were washed, minced, and homogenized in the tissue lysis buffer (Beyotime Biotechnology, Shanghai, China), stored in liquid nitrogen. Expression of BRDT was tested by quantitative reverse transcriptase PCR (“qPCR”) and western blotting assays. The written-informed consent was obtained from each participant. The enrolled patients received no prior chemotherapy before surgeries. The surgically excised normal testis tissue was provided by a written-informed testicular cancer patient administrated at Affiliated Kunshan Hospital of Jiangsu University. Experiments and protocols requiring human tissues and cells were approved by the Ethics Board of Affiliated Kunshan Hospital of Jiangsu University, according to Declaration of Helsinki.

### Cell culture

Ovarian cancer cell line CaOV3 was purchased from the Cell Bank of Shanghai Institute of Biological Science, CAS (Shanghai, China). Cells were cultured in the medium previously described^14^. The normal ovarian epithelial (“OE”) cells were provided by Dr. Bi^14^, which were cultured in MCDB109/M199 medium with 20% fetal bovine serum (FBS). The achieved ovarian cancer tissues were washed in phosphate-buffered saline for five times and minced into small pieces (1 mm^3^), which were digested by Collagenase I (Sigma-Aldrich) and DNase (Sigma-Aldrich). Single-cell suspensions were pelleted and washed in Dulbecco’s modified Eagle medium (DMEM). Fibroblasts, blood vessel cells, and immune cells were abandoned via mechanical means and centrifugation. Purified primary human ovarian cancer cells were cultured in the medium previously described^15^. Four different ovarian cancer cells were established, named “pOC-1/4”. All cells were regularly checked to exclude possible mycoplasma and microbial contamination. Authentication by STR profiling, population doubling time, and cell morphology were also routinely examined to verify the genotypes every 3–4 months.

### qPCR

Trizol reagents (Promega, Madison, WI) were added for RNA extraction. A SYBR Green PCR kit (Applied Biosystems, Foster City, CA) was utilized for reverse transcription under the ABI Prism7500 Fast Real-Time PCR system. A melt curve analysis was performed to calculate product melting temperatures. To quantify targeted gene expression a 2^−∆∆Ct^ method was applied, with *GAPDH* tested as the internal control. The primers were listed in Table 1.

**Table 1:** Sequences utilized in this study.

qPCR primers		
Genes	Forward (5′–3′)	Reverse (5′–3′)
*BRDT*	AAGCCTCCTCTGAAGGGAACTC	GGGACAAAACCTGGAGCTGTTG
*PLK1*	GCACAGTGTCAATGCCTCCAAG	GCCGTACTTGTCCGAATAGTCC
*AURKC*	CCACAGTGAGACTTACAGACGC	GCTGGTATCTGAGAAGCCTGGA
*GAPDH*	GTCTCCTCTGACTTCAACAGCG	ACCACCCTGTTGCTGTAGCCAA
shRNA sequences (5′–3′)		
Scramble shRNA	UAGCGACUAAACACAUCAATT	
BRDT shRNA-1	AAGACTTCAATACAATGTTCTCA	
BRDT shRNA-2	AAGATGAGCGAGTTAAGCGTCTT	
BRDT shRNA-3	AATAATGATGTCCAAAGAAGAAC	
PLK1 shRNA	GTGCTTCGAGATCTCGGAC	
AURKC	CCACGATAATAGAGGAGTTGGCAGATGCC	
sgRNA sequences (5′–3′)		
BRDT sgRNA-1	TCTCCCTTGAACGTGGTACA (Target DNA Sequence)	
BRDT sgRNA-2	TCTCCCTTGAACGTGGTACA (Target DNA Sequence)	

### Western blotting

Cells or tissues were incubated with the described lysis buffer^14^. Aliquots of 40 µg of proteins of each condition were separated by 10% sodium dodecyl sulphate–polyacrylamide gel electrophoresis gels and then transferred to PVDF blots (Millipore, Bedford, MA). After blocking, blots were incubated with specific primary antibodies (overnight at 4 °C) and corresponding secondary antibodies (2 h at room temperature)^14^. Antibody-antigen bindings were tested by an enhanced chemiluminescence (ECL) system (Amersham Biosciences, Piscataway, NJ). An Image J software (NIH) was utilized for data quantification.

### MTT assay

Cells, with different genetic manipulations, were initially seeded into 96-well plates at a density of 2.5 × 10^3^ cells per well. After incubation for 72 h, the cell viability was tested by MTT assay. MTT optical density (OD) was measured at 550 nm.

### BrdU assay

Ovarian cancer cells were seeded into 96-well plates at a density of 2.5 × 10^3^ cells per well. Following incubation with BrdU (10 μM, Cell Signaling Tech, Shanghai, China) for 48 h, cells were washed and BrdU incorporation was tested by an ELISA kit (Cell Signaling Tech), with ELISA absorbance tested at 405 nm.

### “Transwell” assay

Genetically modified CaOV3 cells (1.5 × 10^5^ cells in 250 μL serum-free medium) were seeded into “Transwell” upper chambers (16-μm pore size, BD Biosciences, Shanghai, China). The lower compartments were filled with 10% FBS medium. After 24 h, the non-migrated cells on the upper surface were removed. The migrated cells, on the lower surface, were fixed, stained and counted.

### Caspase-3 activity assay

Ovarian cancer cell lysates (20 μg of each treatment) were mixed with the described assay buffer^16^, together with the caspase-3 substrate (Ac-DEVD-AFC). Following extensive washes, the caspase-3 enzymatic AFC activity was measured at the excitation wavelength of 380 nm and the emission wavelength of 440 nm.

### EdU staining

Ovarian cancer cells with applied genetic modifications were seeded into 24-well plates (3 × 10^4^ cells per well) and cultured for 48 h. Next, an EdU (5-ethynyl-20-deoxyuridine) staining assay was performed using the described protocol elsewhere^17,18^. EdU percentages (EdU vs. DAPI, %) of 800 cells in five random views (per treatment, 1 × 100 magnification) were recorded.

### Mitochondrial depolarization

JC-1 fluorescence dye can form green monomers by aggregating in the mitochondria in apoptotic cells with mitochondrial depolarization^19^. Ovarian cancer cells with applied genetic modifications were seeded into 24-well plates (3 × 10^4^ cells per well) and cultured for 48 h. Cells were then stained with JC-1 (10 μg/mL, Sigma). JC-1 fluorescence images, integrating green (at 488 nm) and red (at 625 nm) wavelengths were presented. JC-1 green fluorescence intensity (at 488 nm) was recorded as well.

### Short-hairpin RNA (shRNA)-mediated gene silencing

There different shRNAs, targeting non-overlapping sequences (“−1/−2/−3”, listed in Table 1) of *BRDT*, were individually sub-cloned into a GV248 (hU6-MCS-Ubiquitin-EGFP-IRES-puromycin) construct. The shRNA construct, along with the lentivirus package plasmids, were co-transfected to HEK-293 cells to generate BRDT shRNA lentivirus. Following filtration and enrichment, the virus was added to ovarian cancer cells (cultured in the polybrene medium, same for all virus procedures). The infected cells were then subjected to selection by puromycin (2.0 μg/mL) for another 4–5 passages. Knockdown of BRDT in the stable cells was verified by qPCR and Western blotting assays. Silencing of PLK1 and AURKC was through the same procedure, with their shRNA sequences listed in Table 1.

### BRDT knockout

The single-guide RNA (sgRNA) targeting BRDT (two different sequences, “sgRNA-1/-2”, listed in Table 1, provided by Shanghai Genechem Co.) was sub-cloned into a lenti-CRISPR-GFP-puro construct, which was transfected to HEK-293 cells along with the lentivirus package plasmids to generate BRDT-KO virus. The virus was filtered, enriched and added to ovarian cancer cells for 24 h. Cells were subjected to selection with puromycin (2.0 μg/mL) for 4–5 passages. In the resulting stable cells BRDT KO was verified by western blotting and qPCR assays.

### Overexpression of BRDT and others

The full-length *BRDT cDNA* was synthesized and sequence-verified by Shanghai Genechem Co, sub-cloned to a GV248 vector. The construct was then transfected to HEK-293 cells with the lentiviral packaging plasmids^20^, generating BRDT-expressing lentivirus (“LV-BRDT”). Following filtration and enrichment, LV-BRDT was added to ovarian cancer cells. Afterwards, puromycin (2.0 μg/mL) was included to select stable cells, where BRDT overexpression was verified by Western blotting and qPCR assays. Control cells were infected with lentivirus with empty vector (“LV-C”). Ectopic overexpression of PLK1 and AURKC was through the same protocol.

### Xenograft assay

The severe combined immunodeficient (SCID) mice (17.5–18.5 g, 4–5-week-old) were obtained from the Animal Center of Chinese Academy of Science (Shanghai, China). CaOV3 or pOC-1 primary cells (for each mouse, 5 × 10^6^ cells in 100 μL DMEM plus 100 μL Matrigel, no serum) were subcutaneously (s.c.) injected to the right flanks of SCID mice. After 3 weeks the subcutaneous xenografts were established (around 100 mm^3^), and recordings were initiated (Day-0, or “D0”). Mice body weights and bi-dimensional tumor measurements were recorded every seven days for total 35 days. The animal protocols were approved by the Ethics Board and IACUC of Affiliated Kunshan Hospital of Jiangsu University.

### Statistical analyses

In vitro experiments were repeated at least three times and similar results were obtained. Values were normalized when necessary and expressed as mean ± standard deviation (SD, normal distribution). For statistical analyses the SPSS software (version 21.0, using one-way ANOVA) was employed. To test significance between two treatment groups, a two-tailed unpaired *t-*test (Excel 2007) was utilized. All differences were considered significant at *P* < 0.05.

## Results

### BRDT overexpression in ovarian cancer

**Fig. 1:**
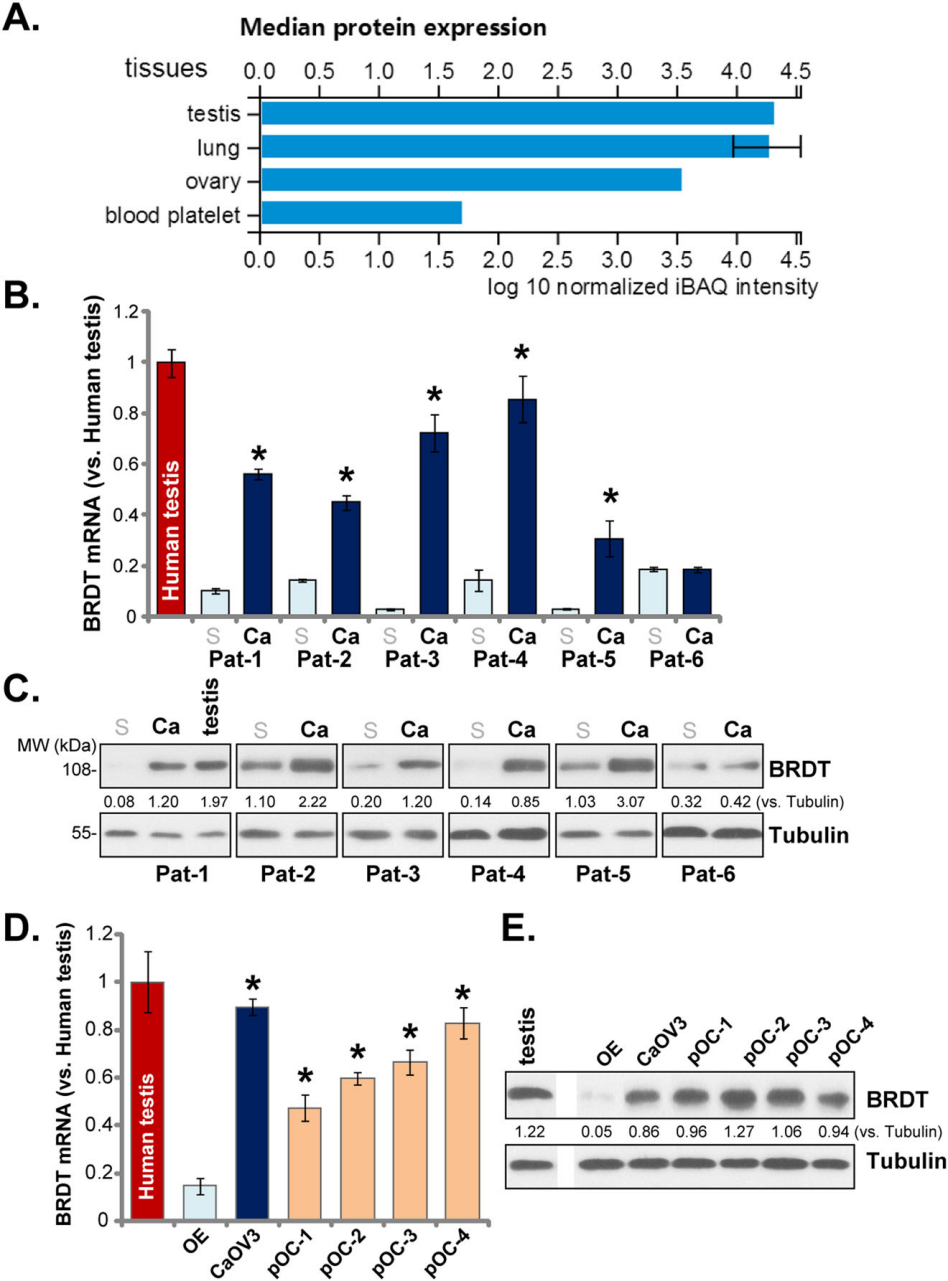
BRDT overexpression in ovarian cancer. BRDT protein expression profile from the proteomicsdb database (**A**). *BRDT* mRNA and protein expression in the listed human ovarian cancer tissues (“Ca”) and para-cancer normal ovarian epithelial tissues (“S”), as well as in the listed ovarian cancer cells and ovarian epithelial (“OE”) cells was tested by qPCR (**B** and **D**) and Western blotting (**C** and **E**) assays. Each tissue was randomly cut into five different pieces (**B**). BRDT expression in human testis tissues was tested as the positive control (**B**–**E**). BRDT protein expression was quantified and normalized to Tubulin (**C** and **E**). For each assay, n = 5 (**D**). *P < 0.05 vs. “S” tissues (**B**) or “OE” cells (**D**). Experiments in this figure were repeated three times, with similar results obtained.

First, the database proteomicsdb (www.proteomicsdb.org) was consulted to verify BRDT expression in human tissues. As shown, BRDT protein is primarily expressed in testis, lung and ovarian tissues (Fig. 1A), very few was detected in other human tissues. To verify the data, a total of six ovarian cancer tissues (“Ca”) and paired surrounding normal ovarian epithelial tissues (“S”) were tested. These tissues were derived from six different primary ovarian cancer patients: Pat-1 to Pat-6. The qPCR results, Fig. 1B, demonstrated that *BRDT* mRNA expression was relatively low in normal ovarian epithelial tissues (Fig. 1B), but was significantly upregulated in five out of six cancer tissues (Pat-1 to Pat-5, Fig. 1B). BRDT protein upregulation was detected as well in the five ovarian cancer tissues (Fig. 1C). Again, low BRDT protein expression was detected in ovarian epithelial tissues (Fig. 1C). BRDT expression in human testis tissue was shown as the positive control (Fig. 1B, C).

In established (CaOV3 cell line) and primary human ovarian cancer cells, *BRDT* mRNA (Fig. 1D) and protein (Fig. 1E) expression was significantly higher than that in ovarian epithelial (“OE”) cells. The primary cancer cells were derived from the four ovarian cancer tissues with significant BRDT upregulation (“Pat-1/-2/-3/-4”, see Fig. 1B, C). These results together show that BRDT is overexpressed in human ovarian cancer tissues and cells.

### BRDT shRNA inhibits ovarian cancer cell survival, growth, proliferation, and migration

**Fig. 2:**
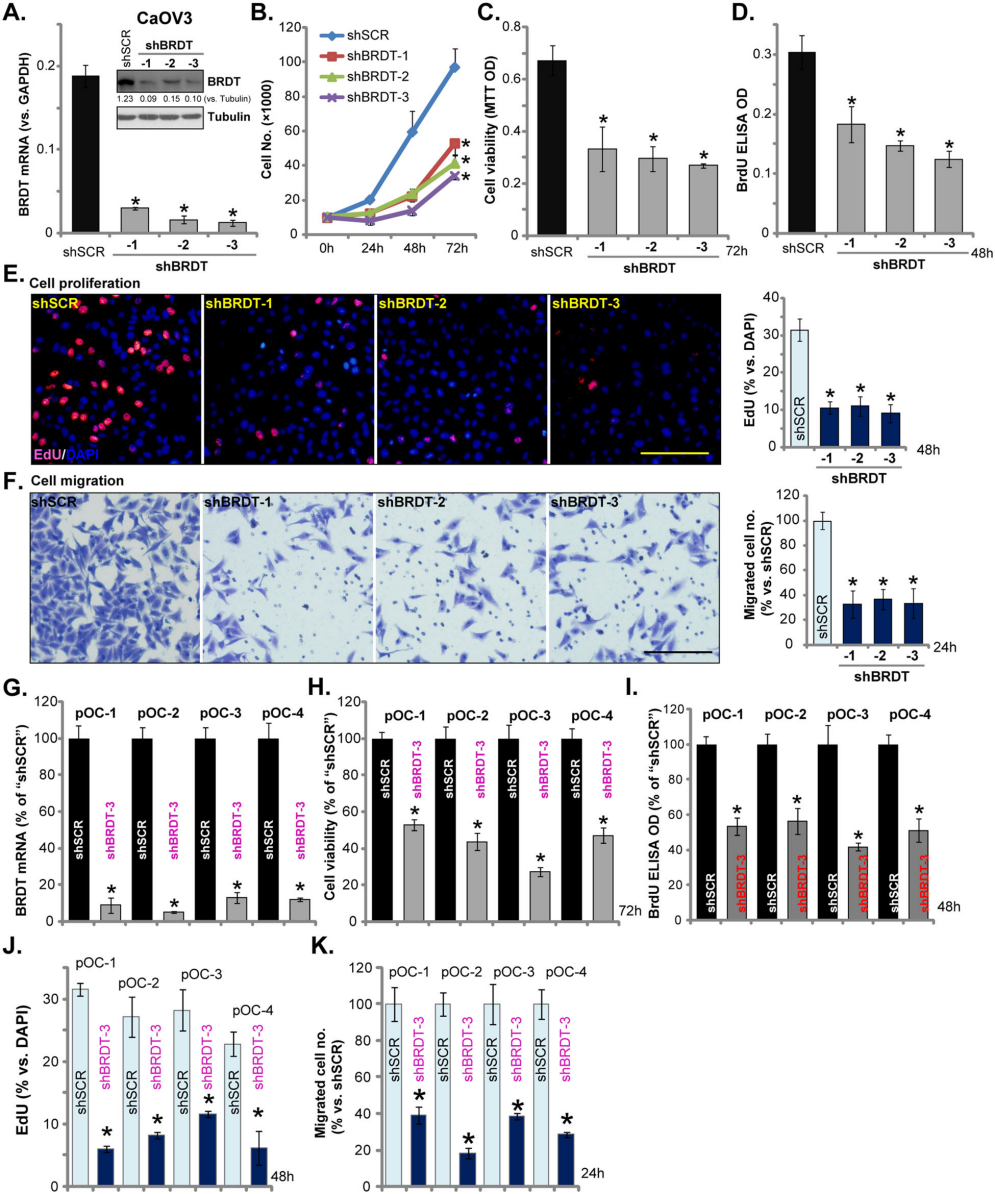
BRDT shRNA inhibits ovarian cancer cell survival, growth, proliferation and migration. CaOV3 cells (**A**–**F**) or the primary human ovarian cancer cells (“pOC-1/-2/-3/-4”) (**G**–**K**) were transfected with scramble control shRNA lentivirus (“shSCR”) or the BRDT shRNA lentivirus (“shBRDT-1/-2/-3”, with different shRNA sequence) for 24 h, stable cells were selected by puromycin. *BRDT* mRNA or protein expression was shown (**A** and **G**); Cell growth (**B**), viability (MTT assay, **C** and **H**), as well as proliferation (BrdU ELISA and EdU staining assays, **D**, **E**, **I** and **J**), and cell migration (“Transwell” assays, **F** and **K**) were tested by the appropriate assays, results were quantified. For the functional assays, the exact same amount of viable cells with different genetic modifications were initially (at 0 h) seeded into each well/dish and cultured for applied time periods (same for all Figures). BRDT protein expression was quantified and normalized to Tubulin (**A**). For each assay, *n* = 5 (five dishes or wells). *P < 0.05 vs. “shSCR” cells. Experiments in this figure were repeated five times, with similar results obtained. Scale Bar = 100 μm (**E** and **F**).

We tested whether BRDT played a role in the oncogenic behaviors of ovarian cancer cells. Three different BRDT shRNAs, with non-overlapping sequences (namely “shBRDT-1/-2/-3”, listed in Table 1), were individually transfected to CaOV3 cells. Following selection by puromycin, stable cells were established. Analyzing *BRDT* mRNA expression, by qPCR, demonstrated that *BRDT mRNA* levels were significantly decreased in stable cells with BRDT shRNA (Fig. 2A). BRDT protein levels were downregulated as well (Fig. 2A). Cell counting assay results, in Fig. 2B, demonstrated that stable CaOV3 cells with BRDT shRNA grew significantly slower than control cells (with scramble control shRNA/“shSCR”). BRDT knockdown in CaOV3 cells inhibited cell viability (MTT OD, Fig. 2C) and proliferation (BrdU incorporation, Fig. 2D). Furthermore, BRDT silencing potently decreased EdU-positive nuclei ratio in CaOV3 cells, further confirming proliferation inhibition (Fig. 2E). “Transwell” assay results, Fig. 2F, demonstrated that the applied BRDT shRNAs inhibited CaOV3 cell migration in vitro (Fig. 2F).

To test BRDT’s function in primary cells, primary human ovarian cancer cells (“pOC-1/-2/-3/-4”, derived from different patients, see Fig. 1) were transfected with the lentiviral BRDT shRNA (“shBRDT-3”) as well. Stable cells were achieved by puromycin selection, showing significantly decreased *BRDT mRNA* expression (Fig. 2G). BRDT shRNA potently inhibited cell viability (MTT OD, Fig. 2H) and proliferation (BrdU ELISA OD, Fig. 2I) in primary cancer cells. Additionally, EdU-positive nuclei ratio was significantly decreased following BRDT silencing (Fig. 2J). Moreover, BRDT silencing inhibited in vitro migration of primary cancer cells (“Transwell” assays, Fig. 2K). Altogether, these results show that BRDT silencing exerted significant anti-ovarian cancer cell activity.

### BRDT silencing provokes apoptosis activation in ovarian cancer cells

**Fig. 3:**
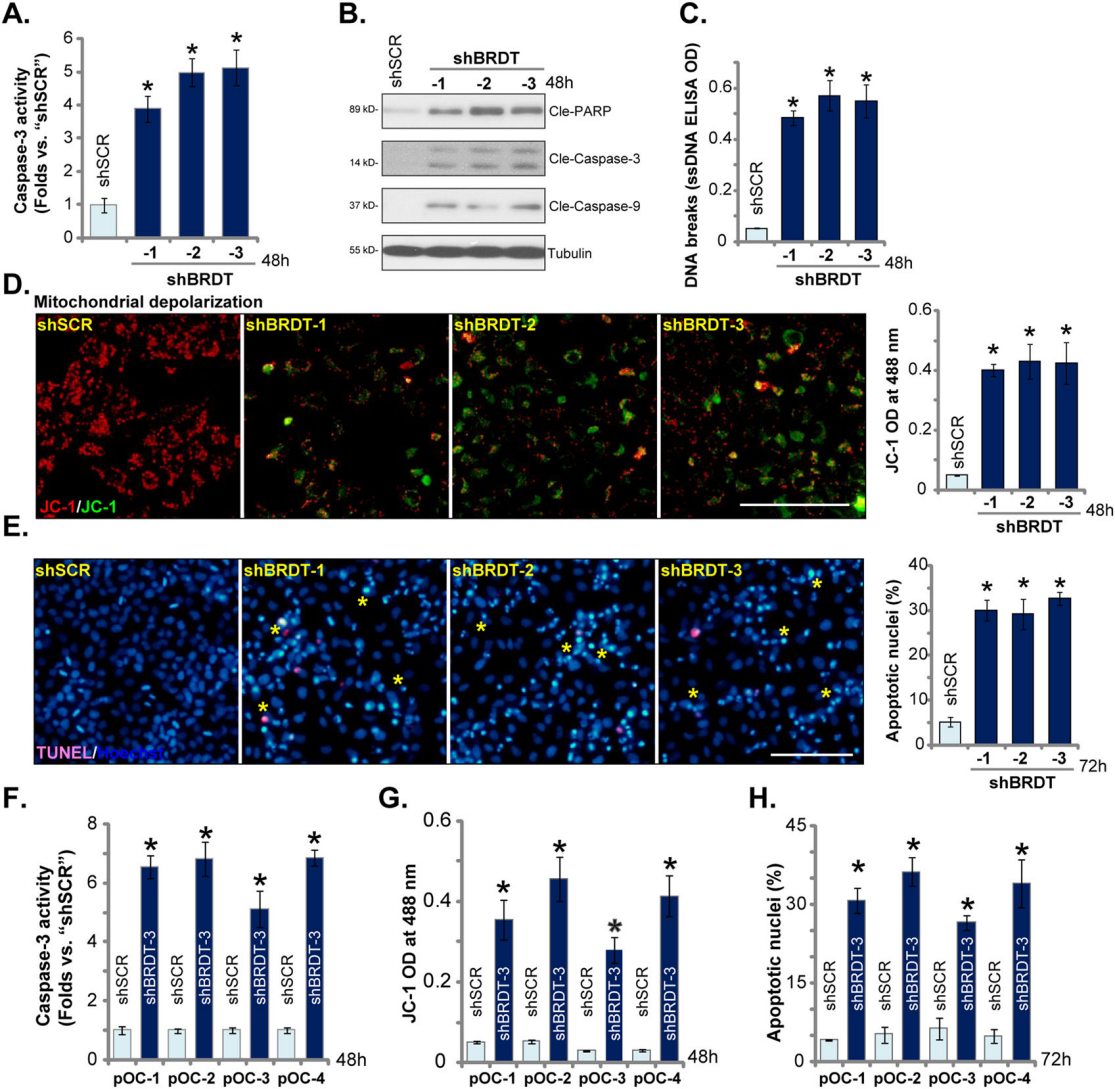
BRDT silencing provokes apoptosis activation in ovarian cancer cells. CaOV3 cells (**A**–**E**) or the primary human ovarian cancer cells (“pOC-1/-2/-3/-4”) (**F**–**H**) were transfected with scramble control shRNA lentivirus (“shSCR”) or the BRDT shRNA lentivirus (“shBRDT-1/-2/-3”, with different shRNA sequence) for 24 h, stable cells were selected by puromycin; Cells were further cultured for applied time periods, caspase activation (**A**, **B**, and **F**), single strand DNA (ssDNA) contents (**C**), mitochondrial depolarization (JC-1 assay, **D** and **G**) were tested. Cell apoptosis was examined by and Hoechst 33342 nuclei staining assay (**E** and **H**). For each assay, n = 5 (five dishes or wells). *P < 0.05 vs. “shSCR” cells. Experiments in this figure were repeated five times, with similar results obtained. Scale Bar = 100 μm (**D** and **E**).

In ovarian cancer cells viability inhibition and growth arrest could induce apoptosis activation^21,22^. Next, we test whether BRDT silencing can induce apoptosis in ovarian cancer cells. We showed that BRDT silencing, by shBRDT-1/-2/-3 (see Fig. 2), significantly increased caspase-3 activity in CaOV3 cells (Fig. 3A). Cleavages of caspase-3, caspase-9 and PARP were detected in BRDT-silenced CaOV3 cells (Fig. 3B). Furthermore, BRDT shRNA-induced DNA breaks, which were evidenced by single strand DNA (ssDNA) accumulation (Fig. 3C). Additional experimental results showed that JC-1 green monomers were accumulated in BRDT-silenced CaOV3 cells, indicating mitochondrial depolarization^19^ (Fig. 3D). These results indicated activation of mitochondrial apoptosis pathway in BRDT-silenced CaOV3 cells^23–26^. In BRDT shRNA-expressing CaOV3 cells, apoptosis activation was further confirmed by increased number of apoptotic nuclei, showing condensed or fragmented nuclear Hoechst 33342 staining (Fig. 3E).The characteristic apoptotic nuclei were labeled with yellow stars (Fig. 3E), although only part of them were TUNEL-positive (purple fluorescence, Fig. 3E, same for all figures).

In the primary human ovarian cancer cells (“pOC-1/-2/-3/-4”), shBRDT-3-induced BRDT silencing (see Fig. 2) resulted in caspase-3 activity increase (Fig. 3F), mitochondrial depolarization (JC-1 green monomers accumulation, Fig. 3G) and apoptotic nuclei increase (Fig. 3H), confirming apoptosis activation. Collectively, these results show that BRDT silencing-induced apoptosis activation in ovarian cancer cells.

### CRISPR/Cas9-induced BRDT knockout potently inhibits ovarian cancer cell progression

**Fig. 4:**
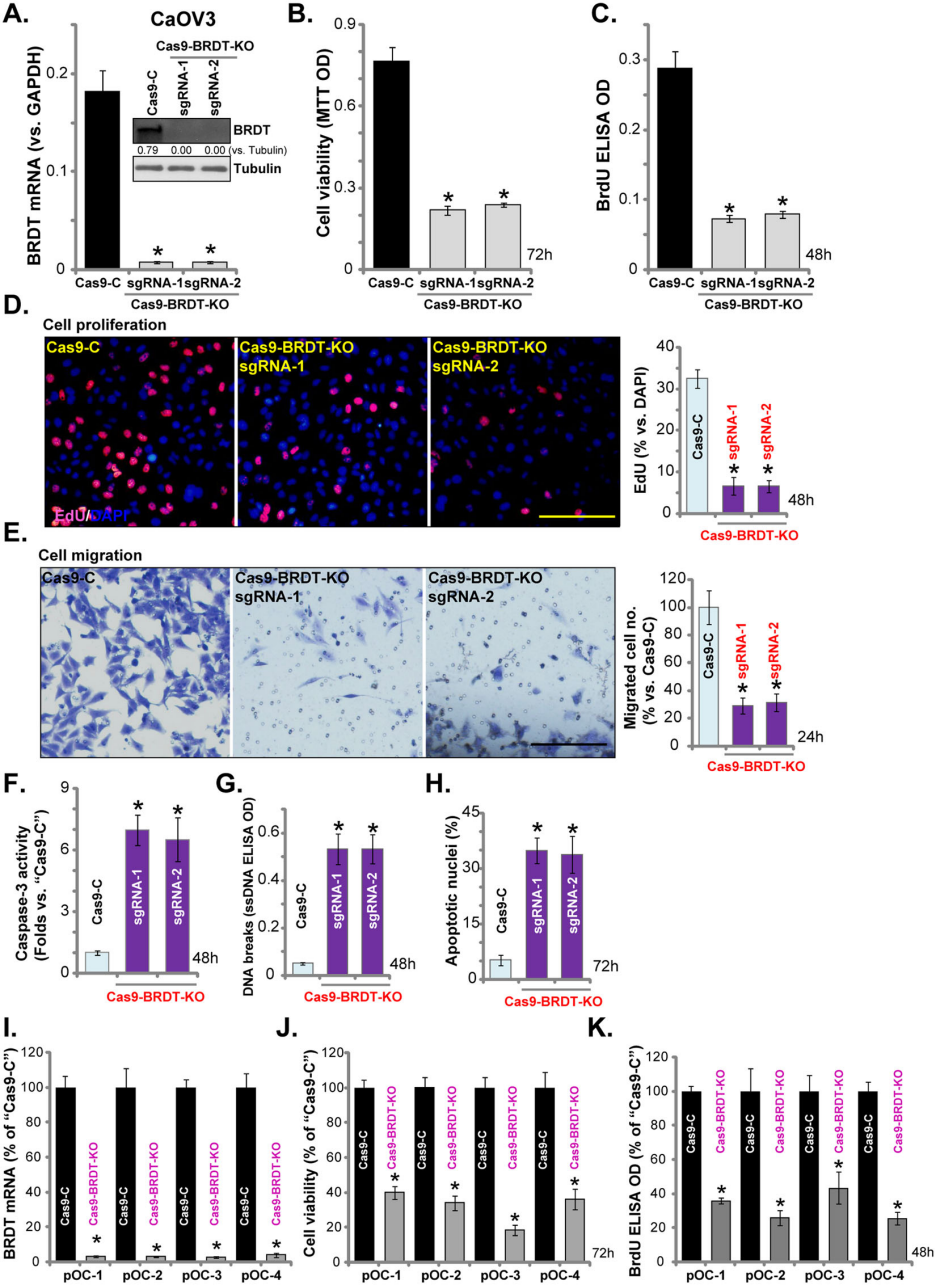
CRISPR/Cas9-induced BRDT knockout potently inhibits ovarian cancer cell progression. CaOV3 cells (**A**–**H**) or the primary human ovarian cancer cells (“pOC-1/-2/-3/-4”) (**I**–**K**) were transfected with the CRISPR/Cas9-BRDT-KO constructs (with “sgRNA-1/-2”) or the CRISPR/Cas9-control construct (“Cas9-C”), with selection by the puromycin stable cells were established. *BRDT* mRNA or protein expression was shown (**A** and **I**); Cell viability (MTT OD, **B** and **J**) and proliferation (BrdU incorporation and EdU staining assays, **C**, **D** and **K**), as well as cell migration (“Transwell” assay, **E**) were tested. Caspase activation (**F**), single strand DNA (ssDNA) contents (**G**) as well as cell apoptosis (Hoechst 333342 staining, **H**) were tested. For each assay, n = 5 (five dishes or wells). *P < 0.05 vs. “Cas9-C” cells. Experiments in this figure were repeated three times, with similar results obtained. Scale Bar = 100 μm (**D** and **E**).

To further confirm the role of BRDT in ovarian cancer cells, we utilized the CRISPR/Cas9 strategy. As described, two lentiviral CRISPR/Cas9-BRDT-KO constructs, with different sgRNAs (“sgRNA-1/-2”), were individually transduced to CaOV3 cells. Following selection by puromycin stable cells were established. Analyzing *BRDT* mRNA and protein expression in the stable cells confirmed that BRDT was completely depleted by the CRISPR/Cas9 constructs (Fig. 4A). Significantly, in CaOV3 cells BRDT KO potently inhibited cell viability (MTT OD, Fig. 4B). BrdU incorporation (Fig. 4C) and EdU-positive nuclei ratio (Fig. 4D) were decreased in BRDT-KO CaOV3 cells, indicating proliferation inhibition. “Transwell” assay results, Fig. 4E, demonstrated that CaOV3 cell migration was largely inhibited with BRDT KO.

Additional studies demonstrated that CRISPR/Cas9-induced BRDT KO increased caspase-3 activity (Fig. 4F) and induced DNA breaks (ssDNA accumulation, Fig. 4G). Furthermore, significant apoptosis activation was detected in BRDT KO CaOV3 cells, evidenced by increased ratio of apoptotic nuclei (Fig. 4H).

In primary human ovarian cancer cells (“pOC-1/-2/-3/-4”), transfection of lentiviral CRISPR/Cas9-BRDT-KO construct (with “sgRNA-1”) depleted *BRDT* mRNA (Fig. 4I), thereby resulting in viability reduction (Fig. 4J) and proliferation inhibition (Fig. 4K). Therefore, similar to the shRNA results, BRDT KO potently inhibited ovarian cancer cell progression in vitro.

### BRDT overexpression promotes ovarian cancer proliferation and migration

**Fig. 5:**
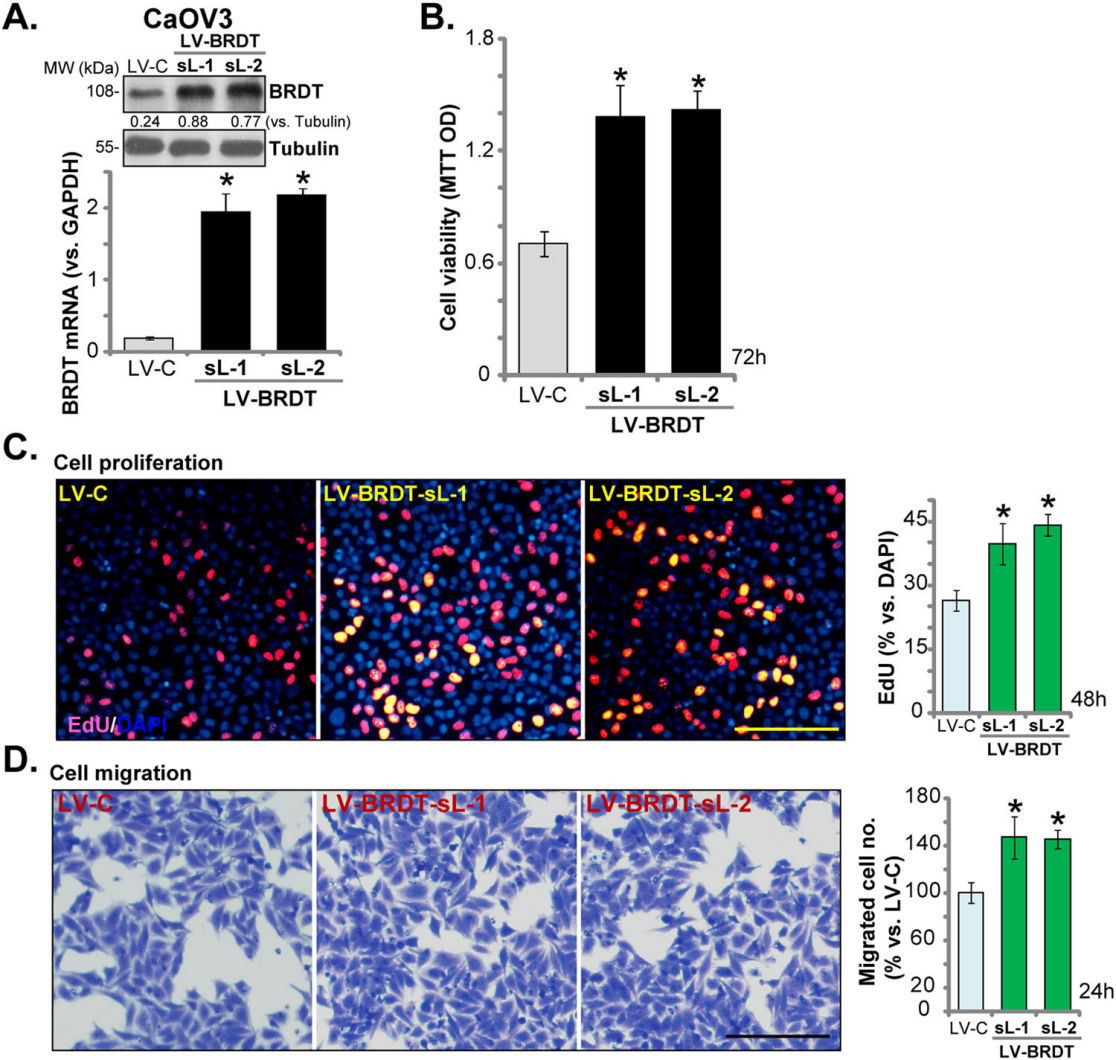
BRDT overexpression promotes ovarian cancer proliferation and migration. CaOV3 cells were transfected with lentiviral BRDT expression construct (“LV-BRDT”) or the empty vector (“LV-C”), stable cells were selected by puromycin. *BRDT* mRNA and protein expression (**A**), cell viability (MTT OD, **B**), proliferation (by recording EdU-positive nuclei ratio, **C**) and migration (“Transwell” assays, **D**) were tested similarly. BRDT protein expression was quantified and normalized to Tubulin (**A**). For each assay, *n* = 5 (five dishes or wells). *P < 0.05 vs. “LV-C” cells. Experiments in this figure were repeated three times, with similar results obtained. Scale Bar = 100 μm (**C** and **D**).

Based on these results, we hypothesized that forced BRDT overexpression might promote ovarian cancer cell growth. To test this hypothesis, a lentiviral BRDT expression construct (“LV-BRDT”) was transfected to CaOV3 cells. Puromycin was applied again to select two stable cell lines (“sL-1/sL-2”). Testing *BRDT* mRNA expression, by qPCR, confirmed that in the LV-BRDT stable cells *BRDT* mRNA levels increased over 10 folds (vs. control cells with empty vector/“LV-C”) (Fig. 5A). BRDT protein levels were increased as well (Fig. 5A). As shown, exogenous BRDT overexpression augmented CaOV3 cell viability (Fig. 5B) and proliferation (by recording EdU-positive nuclei ratio, Fig. 5C). BRDT overexpression also promoted CaOV3 cell migration, tested by “Transwell” assays (Fig. 5D) These results further confirmed that BRDT plays an essential role in ovarian cancer cell progression.

### BRDT depletion-induced anti-ovarian cancer cell activity is associated with PLK1-AURKC downregulation

**Fig. 6:**
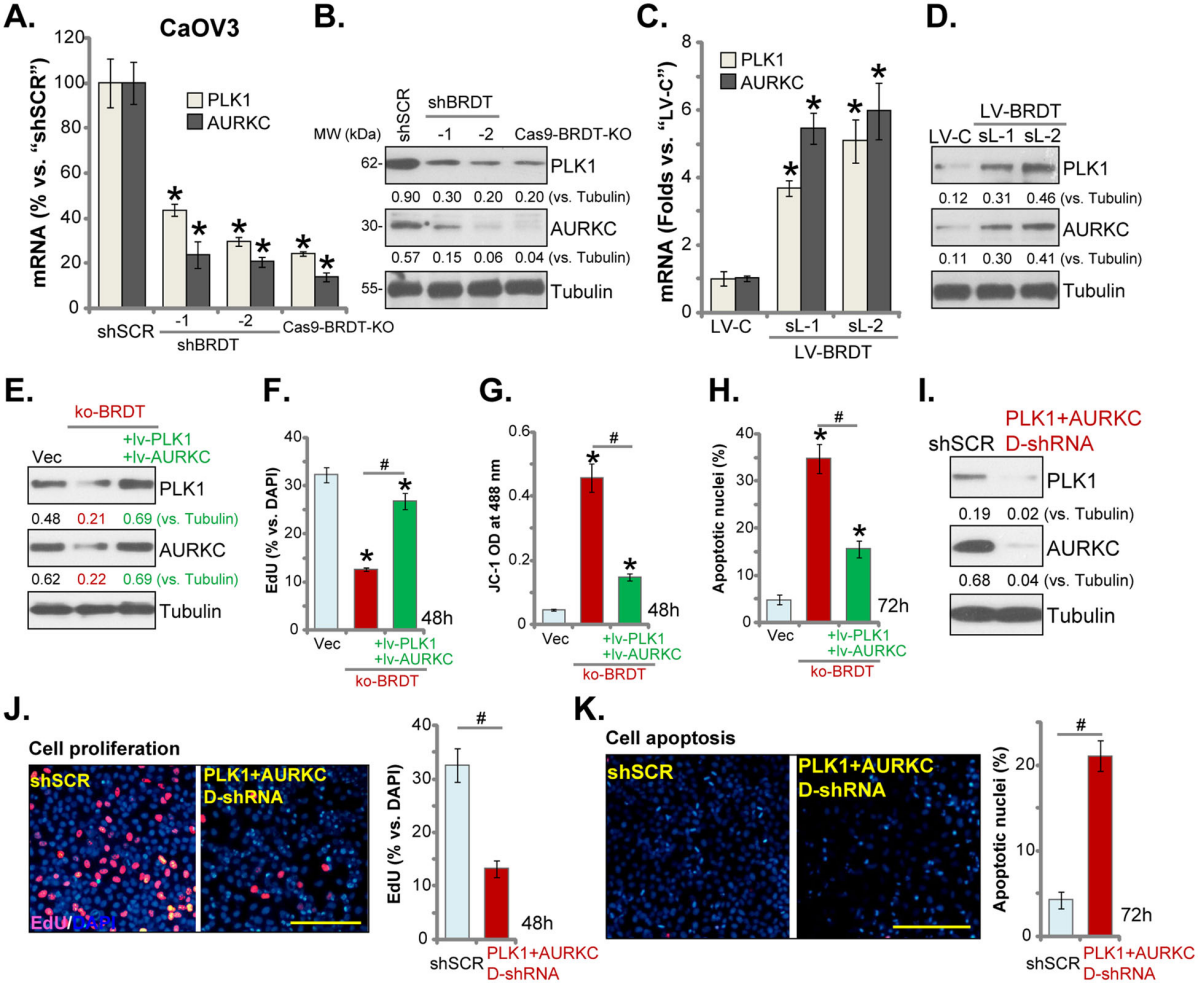
BRDT depletion-induced anti-ovarian cancer cell activity is associated with PLK1-AURKC downregulation. The mRNA and protein expressions of listed genes in the stable CaOV3 cells, with the lentiviral BRDT shRNA (“shBRDT-1/-2”), the lentiviral CRISPR/Cas9-BRDT-KO construct (“sgRNA-1”), or the lentiviral BRDT expression construct (“LV-BRDT”), were tested by qPCR (**A** and **C**) and western blotting (**B** and **D**) assays. Stable CaOV3 cells expressing the CRISPR/Cas9-BRDT-KO construct (with “sgRNA-1”, “ko-BRDT” cells) were further transfected with or without lentivirus encoding PLK1 and AURKC (“ + lv-PLK1 + lv-AURKC”), control cells were with empty vector (“Vec”), expression of listed proteins was shown (**E**); Cells were further cultured for applied time periods, cell proliferation (recording EdU-positive nuclei ratio, **F**), mitochondrial depolarization (JC-1 green monomers intensity, **G**) and apoptosis (recording apoptotic nuclei ratio, **H**) were tested by the appropriate assays. CaOV3 cells were transfected with scramble control shRNA lentivirus (“shSCR”) or PLK1 shRNA lentivirus plus AURKC shRNA lentivirus (“PLK1 + AURKC D-shRNA”) for 24 h, stable cells were selected by puromycin. Expression of listed proteins was shown (**I**). Cells were further cultured for applied time periods, cell proliferation (**J**) and apoptosis (**K**) were tested by EdU staining and apoptotic nuclei staining assays, respectively. For each assay, *n* = 5 (five dishes or wells). *P < 0.05 vs. “shSCR”/“Vec” cells. ^**#**^*P* < 0.05. Expression of listed proteins was quantified and normalized to Tubulin. Experiments in this figure were repeated three times, with similar results obtained. Scale Bar = 100 μm (**J** and **K**).

It has been shown that BRDT is essential for the transcription and expression of PLK1 and AURKC^11^, both are important oncogenic or pro-cancerous genes^13,21,27^. In stable CaOV3 cells with BRDT-shRNA (“shBRDT-1/-2”, see Fig. 2) or CRISPR/Cas9-BRDT-KO construct (“sgRNA-1”, see Fig. 4), mRNA and protein expression of PLK1 and AURKC were significantly downregulated (Fig. 6A, B). Conversely, forced BRDT overexpression in CaOV3 cells (see Fig. 5) significantly increased PLK1 and AURKC expression (both mRNA and protein) (Fig. 6C, D). These results suggest that BRDT is indeed important for PLK1 and AURKC expression in ovarian cancer cells.

To test the link between BRDT depletion-induced anti-ovarian cancer cell activity and PLK1-AURKC downregulation, BRDT-KO CaOV3 cells were further transfected with lentivirus encoding PLK1 and AURKC (“ + lv-PLK1 + lv-AURKC”). Stable cells were established with puromycin selection. As shown PLK1 and AURKC protein expression was restored by the two constructs in BRDT-KO CaOV3 cells (Fig. 6E). Functional studies demonstrated that BRDT-KO-induced proliferation inhibition (EdU ratio decrease, Fig. 6F), mitochondrial depolarization (JC-1 green intensity increase, Fig. 6G) and cell apoptosis (increased apoptotic nuclei ratio, Fig. 6H) were largely attenuated with PLK1 and AURKC re-expression (Fig. 6F–H). Thus, PLK1-AURKC downregulation should be a primary mechanism of BRDT depletion-induced anti-ovarian cancer cell activity. However, single overexpression of PLK1 or AURKC only slightly attenuated BRDT-KO-induced proliferation inhibition (Fig. S1A) and apoptosis activation (Fig. S1B) in CaOV3 cells. Single overexpression-induced rescue effect in the ko-BRDT cells was significantly weaker than double overexpression (Fig. 6F–H).

To further support our hypothesis, CaOV3 cells were co-transfected with lentivirus encoding PLK1 shRNA and AURKC shRNA. Stable cells were again achieved: namely “PLK1 + AURKC D-shRNA” cells. As shown PLK1 and AURKC protein expression was significantly downregulated in CaOV3 cells with “PLK1 + AURKC D-shRNA” (Fig. 6I), where significant proliferation inhibition (decreased nuclear EdU ratio, Fig. 6J) and apoptosis (Fig. 6K) were detected.

Notably, shRNA-induced single knockdown of PLK1 or AURKC only slightly induced proliferation inhibition (by recording EdU-positive nuclei ratio, Fig. S1C) and apoptosis activation (Fig. S1D). Importantly, PLK1 plus AURKC double knockdown (“D-shRNA”)-induced anti-CaOV3 cell activity was stronger than single knockdown (Fig. S1C, D), but was weaker than BRDT KO (Fig. S1C, D). These results implied that other protein targets, besides PLK1 or AURKC, should also participate in BRDT-mediated ovarian cancer cell progression. qPCR assay results, Figure S1E, demonstrated expression of targeted mRNAs in CaOV3 cells with applied genetic modifications.

### BRDT silencing inhibits ovarian cancer xenograft growth in SCID mice

**Fig. 7:**
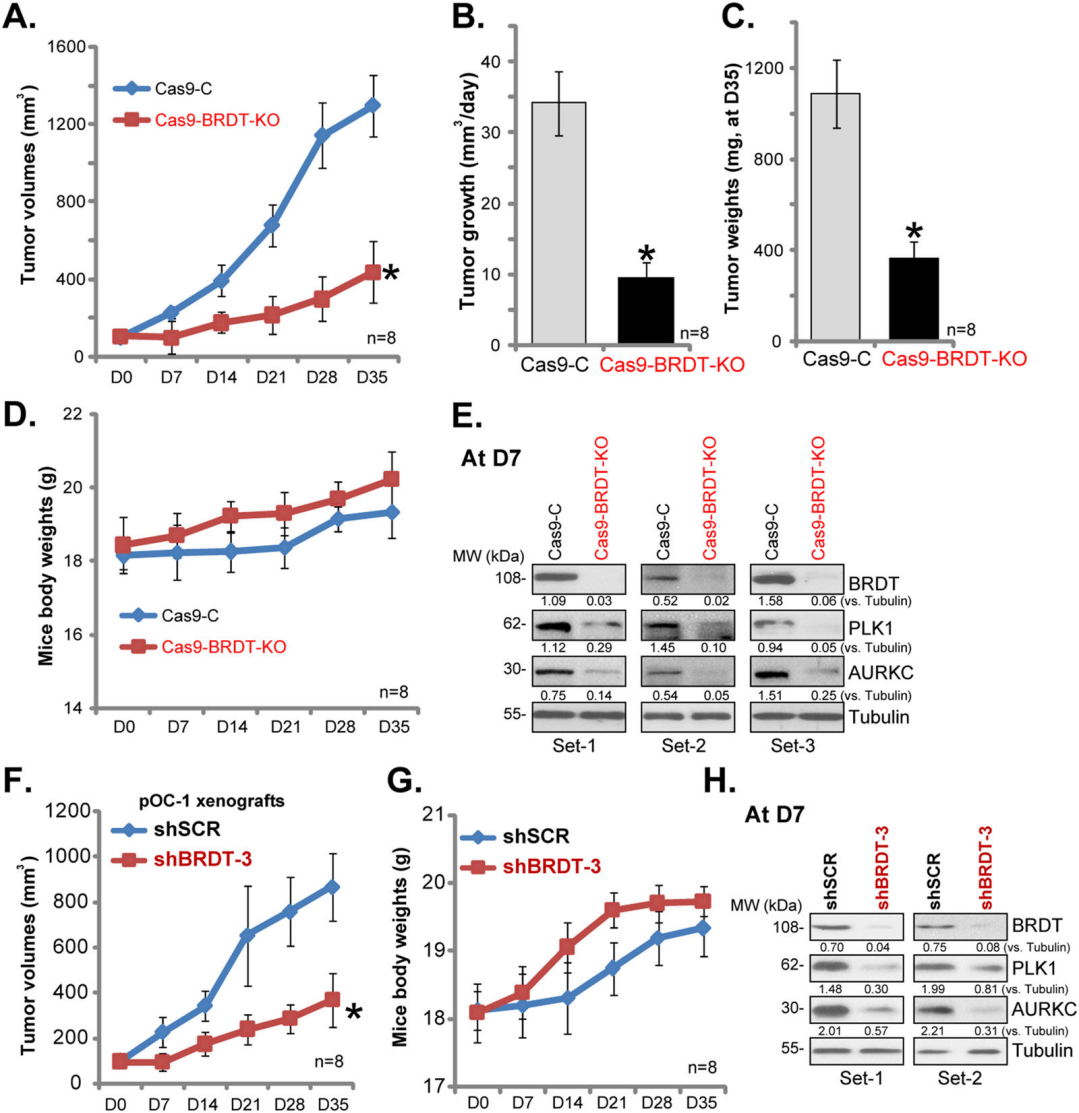
BRDT silencing inhibits ovarian cancer xenograft growth in SCID mice. The SCID mice were injected s.c. with control and BRDT-KO CaOV3 cells (5 × 10^6^ cells in 100 μL DMEM plus 100 μL Matrigel, no serum) at the right flanks, within 3 weeks the xenografts were established (Day-0, “D0”). The tumor volumes (**A**) and mice body weights (**B**) were recorded every seven days for total 35 days; The estimated daily tumor growth was calculated (**C**); At D35 all tumors were isolated and weighted (**D**). At D7 three tumors of each group were isolated and tissues lysates were subjected to Western blotting assays of listed proteins (**E**). The SCID mice were injected s.c. with pOC-1 primary ovarian cancer cells (5 × 10^6^ cells in 100 μL DMEM plus 100 μL Matrigel, no serum) at the right flanks. Within 3 weeks the xenografts were established (Day-0, “D0”). The mice were then subjected to intratumoral injection of either BRDT shRNA lentivirus (“shBRDT-3”) or control shRNA lentivirus (shSCR). Thereafter, the tumor volumes (**F**) and mice body weights (**G**) were recorded every 7 days; At Day-7/D7, two tumors of each group were isolated and tissues lysates were subjected to Western blotting assays (**H**). Listed protein expression was quantified and normalized to Tubulin (**E** and **H**). *n* = 8 mice per group *P < 0.05 vs. “Cas9-C”/“shSCR” tumors.

In order to study the potential effect of BRDT in ovarian cancer cell growth in vivo, control CaOV3 cells (“Cas9-C”) and BRDT-KO (with “sgRNA-1”) CaOV3 cells were inoculated to SCID mice to form subcutaneous xenografts. Tumor growth curve results, Fig. 7A, demonstrated that xenografts-derived from BRDT-KO CaOV3 cells grew significantly slower than the control tumors (formed by Cas9-C cells). By calculating the estimated daily tumor growth, using the formula [tumor volume at Day-35 (D35) subtracting tumor volume at Day-0 (D0)]/35, we show that BRDT-KO CaOV3 tumor growth was significantly inhibited (Fig. 7B). At D35 tumors of the two groups were isolated and weighted. As demonstrated, BRDT-KO CaOV3 xenografts were significantly lighter than the control tumors (Fig. 7C). The mice body weights, on the other hand, were not significantly different between the two groups (Fig. 7D). There were no noticeable signs of apparent toxicity. These results suggest that BRDT-KO significantly inhibited CaOV3 cell growth in vivo.

To testing signaling changes, at D7, three tumors of each group were isolated and tissues lysates were analyzed for signaling proteins. As shown, BRDT was depleted in BRDT-KO CaOV3 xenografts (Fig. 7E), where PLK1 and AURKC expression was significantly decreased (Fig. 7E). These signaling results in vivo are in line with the in vitro findings.

To further support the role of BRDT in ovarian cancer cell growth in vivo, primary ovarian cancer cells, pOC-1, were s.c. injected to the flanks of SCID mice, forming pOC-1 xenografts within 3 weeks. The mice were then randomly assigned into two groups, receiving intratumoral injection of either BRDT shRNA lentivirus (“shBRDT-3”) or control shRNA lentivirus (shSCR). When recording tumor growth, we found that shBRDT-3 injection potently inhibited pOC-1 xenograft growth in mice (Fig. 7F), but without affecting mice body weights (Fig. 7G). At Day-7 (D7), two tumors of each group were isolated and tissues lysates were subjected to Western blotting assays. Results confirmed BRDT silencing in shBRDT-3-injected tumors (Fig. 7H), with PLK1 and AURKC downregulation observed (Fig. 7H). These results further indicated that BRDT is important for ovarian cancer cell growth in vivo.

## Discussion

BRDT is a driver of meiotic and post-meiotic gene expression^6,28^. BRDT binds to acetylated lysines to regulate epigenetic processes, essential for chromatin structure formation in mitosis progression^6^. Furthermore, BRDT recruits positive transcription elongation factor b (p-TEFb), regulating transcription elongation and expression of several key proteins^6^, including PLK1 and AURKC^11^. A threefold higher BRDT expression is detected in adult testis than that in the embryo testis^29,30^. BRDT expression is correlated with histone H4 hyperacetylation during spermiogenesis^29,30^. In patient with abnormal spermatogenesis, few or no BRDT expression is detected in testis^29,30^. Recent studies have shown that BRDT is reactivated and expressed in various human cancers, including non-small cell lung cancer (NSCLC), head and neck squamous cell carcinomas (HNSCC) and esophagus squamous cell carcinomas, but not in melanoma or in cancers of the colon, breast, kidney and bladder^6,28,30^. The proteomicsdb database and our results in human tissues confirmed that BRDT is expressed in ovarian tissues.

Our results here suggest that BRDT is possibly one important oncogenic gene and therapeutic target of ovarian cancer. Its expression is significantly upregulated in ovarian cancer tissues and in established (CaOV3)/primary human ovarian cancer cells. Low BRDT expression, however, is detected in ovarian epithelial tissues and cells. In ovarian cancer cells, BRDT shRNA or CRISPR-Cas9-indueced BRDT knockout potently inhibited cell growth, survival, proliferation and migration, whereas inducing apoptosis activation. Conversely, forced BRDT overexpression augmented CaOV3 cell proliferation and migration. Importantly, BRDT-KO CaOV3 xenograft tumors grew significantly slower than the control tumors. Furthermore, intratumoral injection of BRDT shRNA lentivirus potently inhibited pOS-1 xenograft growth in SCID mice. These results suggest that targeting BRDT could be a novel and efficient strategy to inhibit ovarian cancer cells, in vitro and in vivo.

PLK1 plays an essential role in regulating cell cycle progression^12^. It is required for a number of cell cycle processes, including cell mitotic entry and G2/M checkpoint, centrosome coordination, spindle assembly and chromosome segregation^12^. PLK1 also exerts key functions at the spindle midzone during abscission, facilitating DNA replication and cytokinesis^12^. It is, therefore, vital for cell cycle progression and cell proliferation^12^. Studies have indicated an oncogenic activity of overexpressed PLK1 in ovarian cancer^21,27^, that is important for cancer cell progression and chemoresistance^21,27^. Additionally, AURKC, another BRDT-regulated gene, is reactivated and overexpressed in multiple different human cancer cells^13^, essential for cancerous behaviors, including cell proliferation and migration^13^. AURKC could promote xenograft tumor growth^13^. Kinase-dead AURKC inhibited HeLa cell proliferation, whereas constitutively active AURKC promoted cancer cell progression^31–33^.

The results of the current study show that PLK1 and AURKC are expressed in ovarian cancer cells. Significantly, the two were downregulated by BRDT shRNA or KO, but being upregulated with ectopic BRDT overexpression. PLK1 and AURKC downregulation was also detected in BRDT-KO CaOV3 tumor tissues and in pOS-1 xenografts with BRDT shRNA injection. Importantly, BRDT-KO-induced anti-ovarian cancer cell activity was largely attenuated with PLK1 and AURKC re-expression. PLK1 plus AURKC double silencing-induced proliferation inhibition and apoptosis activation in CaOV3 cells, mimicking BRDT silencing-induced actions. Therefore, BRDT-mediated ovarian cancer cell progression is associated with regulation of PLK1 and AURKC expression. The detailed mechanisms may warrant further characterizations.

## Conclusion

The current treatments for ovarian cancer, with combination of platinum-based chemotherapy and surgery, can only result in a 5-year overall survival close to 45%. It goes down to 25% for the advanced cancers^34^. The poor survival highlights the limitations in the biological understanding of this devastating disease^35–38^. It is, therefore, urgent to indentify novel therapeutic strategies/molecular targets^35–38^. Recent genomic studies have demonstrated the extensive alternations of epigenetic regulators in ovarian cancers, which might be utilized as basis for developing new epigenetic drugs^35–38^. Our results indicate that BRDT overexpression promotes ovarian cancer cell progression in vitro and in vivo. Targeting BRDT could be a novel strategy to treat ovarian cancer.

## Supplementary information

Supplementary Figure 1
