# Supplementary material for: BRDT promotes ovarian cancer cell growth
Source: Cell Death Dis. 2020 Nov 30;11(11):1021. doi: 10.1038/s41419-020-03225-y (PMC7705741; doi:10.1038/s41419-020-03225-y)
Supplement: Supplementary file 1 — Supplementary Figure 1 [file 41419_2020_3225_MOESM1_ESM.pdf]

## Figure S1

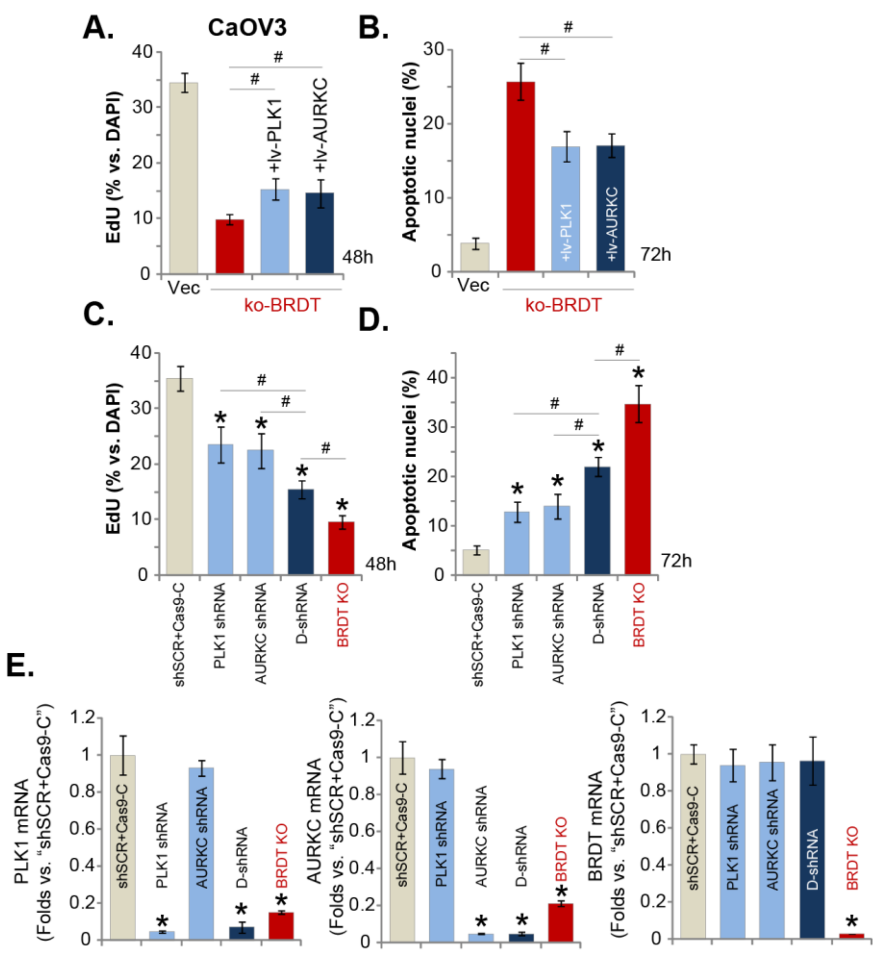


**Figure S1.** CaOV3 cells expressing the CRISPR/Cas9-BRDT-KO construct (with "sgRNA-1", "ko-BRDT" cells) were further transfected with PLK1-expressing lentivirus ("+lv-PLK1") or AURKC-expressing lentivirus ("+lv-AURKC"), stable cells were established. Control cells were with empty vector ("Vec"); Cells were further cultured for applied time periods, cell proliferation (EdU assays, **A**) and apoptosis (**B**) were tested. CaOV3 cells were transfected with PLK1 shRNA lentivirus ("PLK1-shRNA"), AURKC shRNA lentivirus ("AURKC-shRNA") or both ("D-shRNA") for 24h, stable cells were selected by puromycin. These cells, the "ko-BRDT" cells and control cells with scramble control shRNA plus Cas9-C empty vector ("shSCR+Cas9-C") were cultured for applied time periods, cell proliferation (EdU assays, **C**) and apoptosis (**D**) were tested. Expression of listed mRNAs was shown (**E**). For each assay, n=5 (five dishes or wells). ****P*** $<0.05$ *vs.* "shSCR+Cas9-C" cells (**C**-**E**). #***P*** $<0.05$. Experiments in this figure were repeated three times, with similar results obtained.
